# Cepharanthine Enhances TRAIL-Mediated Apoptosis Through STAMBPL1-Mediated Downregulation of Survivin Expression in Renal Carcinoma Cells

**DOI:** 10.3390/ijms19103280

**Published:** 2018-10-22

**Authors:** Sk Abrar Shahriyar, Seon Min Woo, Seung Un Seo, Kyoung-jin Min, Taeg Kyu Kwon

**Affiliations:** Department of Immunology, School of Medicine, Keimyung University, Daegu 42601, Korea; Sksy.kmu@gmail.com (S.A.S.); woosm724@gmail.com (S.M.W.); sbr2010@hanmail.net (S.U.S.); kyoungjin.min@gmail.com (K.-j.M.)

**Keywords:** cepharanthine, TRAIL, survivin, STAMBPL1, DR5, apoptosis

## Abstract

Cepharanthine (CEP) is a natural plant alkaloid, and has anti-inflammatory, antineoplastic, antioxidative and anticancer properties. In this study, we investigated whether CEP could sensitize renal carcinoma Caki cells to tumor necrosis factor-related apoptosis-inducing ligand (TRAIL)-induced apoptosis. CEP alone and TRAIL alone had no effect on apoptosis. However, combined CEP and TRAIL treatment markedly enhanced apoptotic cell death in cancer cells, but not in normal cells. CEP induced downregulation of survivin and cellular-FLICE inhibitory protein (c-FLIP) expression at post-translational levels. Ectopic expression of survivin blocked apoptosis by combined treatment with CEP plus TRAIL, but not in c-FLIP overexpression. Interestingly, CEP induced survivin downregulation through downregulation of deubiquitin protein of STAM-binding protein-like 1 (STAMBPL1). Overexpression of STAMBPL1 markedly recovered CEP-mediated survivin downregulation. Taken together, our study suggests that CEP sensitizes TRAIL-mediated apoptosis through downregulation of survivin expression at the post-translational levels in renal carcinoma cells.

## 1. Introduction

Renal cell carcinoma (RCC) is a major renal neoplasm, and 30% of RCC patients had metastatic disease [1]. Metastatic RCC (mRCC) is fatal, with average survival of only 18 months, and accounts for 2–3% of all adult malignancies [2,3,4]. There are a few drugs with exhibited clinical efficacy in management of metastatic RCC, but the clinical outcomes are not compelling. Although the survival in patients with metastatic RCC has significantly increased by several molecular targeted agents, such as kinase inhibitor and checkpoint inhibitors, those are transitory [5,6,7]. Therefore, unique and efficient therapies are highly needed, and integrated therapies may result in increased efficacy to overcome metastatic RCC compared with single therapies.

Tumor necrosis factor (TNF)-related apoptosis-inducing ligand (TRAIL) has been shown to induce apoptosis in variety of cancer cells, but not most normal human cells [8,9,10,11]. On the contrary, some studies have demonstrated that carcinomas, including malignant RCC, do not exhibit TRAIL-induced apoptosis [7]. Thus, it may not be satisfactory to use TRAIL treatment for numerous malignant tumor cells. Understanding the molecular mechanisms of TRAIL resistance could be used to increase TRAIL sensitivity in cancer cells.

Cepharanthine (CEP; 6′,12′-dimethoxy-2-2′-dimethyl-6,7[methylenebis(oxy)]oxyacanthan) is a biscoclaurine alkaloid which is isolated from the plant *Stephania cepharantha* Hayata. CEP possesses a significant number of biological activities, including antioxidant activity [12,13]. Recently, CEP (10–20 µM) has been established as a potent anticancer drug by abolishing proliferation, cell cycle progression, and malignant tumor invasion in many types of cancer cells [14,15,16]. CEP (2–20 µM) also induces apoptotic cell death through production of reactive oxygen species (ROS) and inhibition of cellular signaling molecules, including NF-κB, STAT3, and JNK [14,17,18,19,20].

Our results showed that CEP (10–15 µM) TRAIL-induced apoptosis and demonstrated the molecular mechanisms in combined treatment-induced apoptosis in renal carcinoma cells.

## 2. Results

### 2.1. CEP Sensitizes TRAIL-Induced Apoptosis in Human Renal Carcinoma Caki Cells

**Figure 1:**
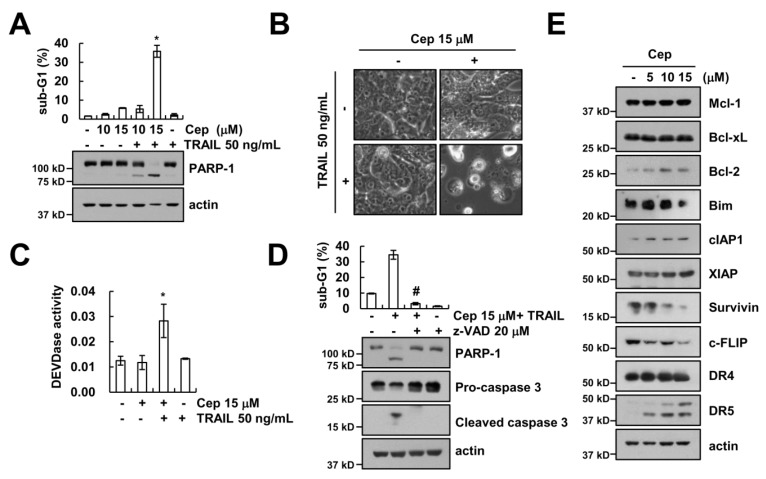
CEP sensitizes TRAIL-mediated apoptosis in human renal carcinoma Caki cells. (**A**) Caki cells were treated with 50 ng/mL TRAIL and/or CEP (10 and 15 µM) for 18 h; (**B**) the photos represent the cellular morphology; (**C**) the graph represents caspase activities; (**D**) Caki cells were pretreated with of 20 µM z-VAD for 30 min, and then 15 µM CEP plus 50 ng/mL TRAIL was added for 18 h; (**E**) Caki cells were treated with 5–15 µM CEP for 18 h. The sub-G1 population was detected by flow cytometry. The protein levels were determined by Western blotting. Data represent the mean ± SD of at least three independent experiments. * *p*  <  0.05 compared with the control, # *p* < 0.05 compared with combined treatment CEP plus TRAIL.

We examined the effect of CEP on TRAIL sensitization in metastatic renal cell carcinoma Caki cells. Cells were treated with CEP alone (10 or 15 µM), TRAIL alone (50 ng/mL), or a combined treatment with CEP and TRAIL. CEP plus TRAIL increased the sub-G1 population and PARP-1 cleavage, whereas CEP alone and TRAIL alone had no effect on cell death (Figure 1A). We fixed the CEP concentration to 15 µM for further study. CEP plus TRAIL enhanced the apoptotic cell morphologies (Figure 1B). Combined CEP and TRAIL treatment induced caspase-3 activation (Figure 1C). To further address the caspase activation in combined treatment-induced apoptosis, we used a pan-caspase inhibitor (z-VAD). z-VAD markedly blocked CEP plus TRAIL-induced apoptosis, PARP-1 cleavage, and cleavage of caspase-3 (Figure 1D). Then, we investigated the fundamental molecular mechanism in Caki cell death by CEP plus TRAIL treatment. CEP induced upregulation of DR5 expression and downregulation of c-FLIP and survivin expression (Figure 1E). However, other apoptotic related proteins (Mcl-1, Bcl-xL, Bcl-2, Bim, cIAP1, DR4, and XIAP) were not altered (Figure 1E). Collectively, these results suggest that CEP plus TRAIL-induced cell death is a caspase-dependent form of apoptosis in human renal cell carcinoma.

### 2.2. CEP Did Not Increase DR5 Expression on the Cell Surface

**Figure 2:**
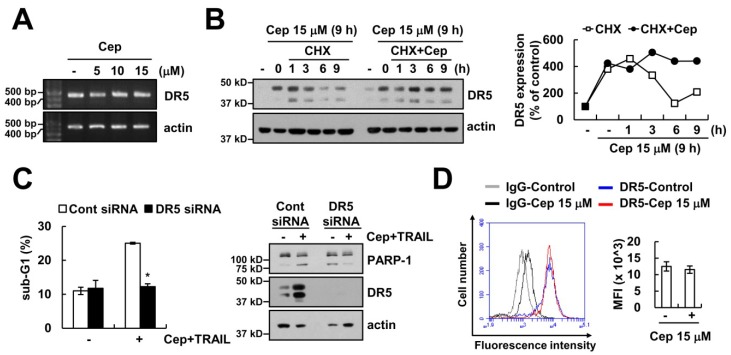
CEP increased DR5 protein expression in the cytosol, but not in the cell surface. (**A**) Caki cells were treated with 5–15 µM CEP for 18 h. DR5 and actin mRNA expression were analyzed by RT-PCR; (**B**) Caki cells were treated with 15 µM CEP for 9 h, and then washed with PBS. After washing, cells were treated with 20 ng/mL cycloheximide (CHX) with or without of 15 µM CEP for 1–9 h The DR5 and actin expression was detected by Western blot (**left panel**). The public domain JAVA image-processing program ImageJ was used for measuring band intensity of DR5 protein (**right panel**); (**C**) Caki cells were transfected control or DR5 siRNA, and then cells were treated with 15 µM CEP plus 50 ng/mL TRAIL for 24 h; (**D**) after treatment with 15 µM CEP for 18 h, DR5 surface expression was determined by flow cytometry. Data represent the mean ± SD of at least three independent experiments. * *p* < 0.05 compared with CEP plus TRAIL-treated control siRNA.

As shown in Figure 1E, CEP increased the protein expression levels of DR5. However, CEP did not alter mRNA levels (Figure 2A). Then, we examined the effect of CEP on DR5 protein stability in Caki cells. After 12 h of CEP treatment, cells were treated by a de novo protein synthesis inhibitor cycloheximide (CHX), in the presence or absence of CEP. CHX in the absence of CEP rapidly downregulated expression of DR5, compared with CHX plus CEP (Figure 2B). To evaluate the role of DR5 upregulation in CEP plus TRAIL-induced apoptosis, we used DR5 small interfering RNA (siRNA). DR5 siRNA-mediated downregulation of DR5 markedly inhibited apoptosis and PARP-1 cleavage (Figure 2C). Unexpectedly, CEP did not increase DR5 surface expression levels (Figure 2D). These data suggest that CEP increased DR5 protein expression in the cytosol, but not in the cell surface. Therefore, DR5 upregulation by CEP might not be critical for CEP-mediated TRAIL sensitization.

### 2.3. CEP Induced Downregulation of c-FLIP Protein Expression

**Figure 3:**
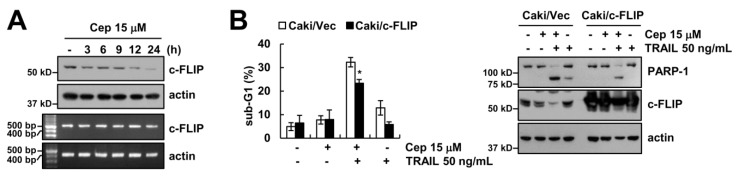
Downregulation of c-FLIP is partially involved in CEP plus TRAIL-induced apoptosis. (**A**) Caki cells were treated with 15 µM CEP for 3–24 h. The c-FLIP expression levels were determined by Western blotting and RT-PCR; (**B**) Vector cells (Caki/Vec) and c-FLIP-overexpressed cells (Caki/c-FLIP) were treated with 15 µM CEP and/or 50 ng/mL TRAIL for 18 h. The sub-G1 population and protein levels were detected by flow cytometry and Western blotting, respectively. Data represent the mean ± SD of at least three independent experiments. * *p*  <  0.05 compared with the CEP plus TRAIL-treated Caki/Vec.

Furthermore, we probed how the expression level of c-FLIP is minimized by CEP. CEP downregulated the c-FLIP protein expression levels in a dose- and time-dependent manner (Figure 1E and Figure 3A), but not its mRNA levels. To investigate the functional importance of c-FLIP protein in the combined treatment, we employed an ectopic expression system. Overexpression of c-FLIP at least partially inhibited CEP plus TRAIL-induced apoptosis and PARP-1 cleavage (Figure 3B). Our data suggests that downregulation of c-FLIP by CEP is partially involved in apoptosis by combined treatment.

### 2.4. Downregulation of Survivin by CEP is Critical to Sensitization of TRAIL-Mediated Apoptosis

**Figure 4:**
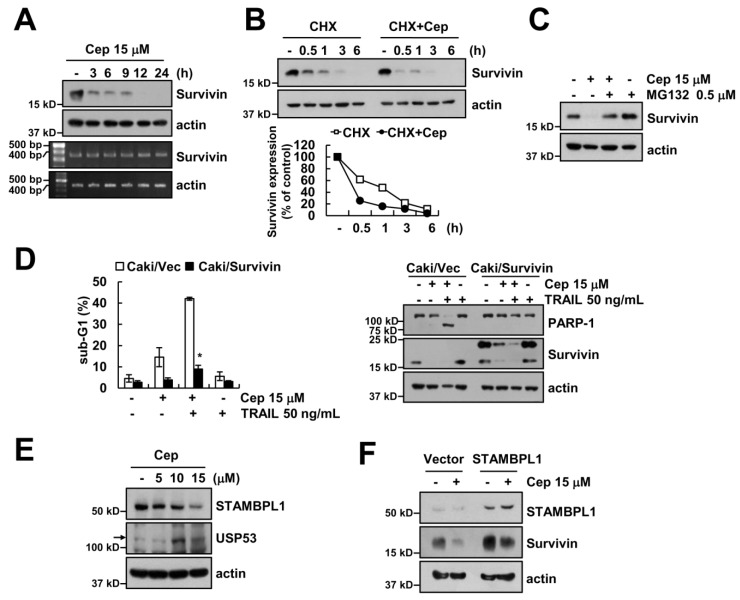
Downregulation of survivin is involved in CEP plus TRAIL-induced apoptosis. (**A**) Caki cells were treated with 15 µM CEP for 3–24 h. The survivin expression levels were determined by Western blotting and RT-PCR; (**B**) Caki cells were treated with 20 ng/mL CHX alone, or CHX plus CEP for 0.5–6 h. The survivin protein band intensity was quantified using ImageJ (public domain JAVA image processing program); (**C**) after pretreatment with 0.5 µM MG132 for 30 min, Caki cells were treated with 15 µM CEP for 18 h; (**D**) vector-transfected cells (Caki/Vec) and survivin-overexpressing cells (Caki/Survivin) were treated with 15 µM CEP and/or 50 ng/mL TRAIL for 18 h. (**E**) Caki cells were treated with CEP (5–15 µM) for 18 h; (**F**) Caki cells were transfected with control or STAMBPL1 expression vector, and then cells were treated with 15 µM CEP for 18 h. The sub-G1 population and protein levels were detected by flow cytometry and Western blotting, respectively. Data represent the mean ± SD of at least three independent experiments. * *p*  <  0.05 compared with the CEP plus TRAIL-treated Caki/Vec.

CEP induced downregulation of survivin protein expression in a time-dependent manner, but not its mRNA expression (Figure 4A). Survivin protein stability remarkably diminished by the treatment of CHX in the presence of CEP (Figure 4B). Next, we examined whether proteasome inhibitor (MG132) reverses CEP-induced c-FLIP downregulation. MG132 prevented c-FLIP downregulation by CEP treatment (Figure 4C). Furthermore, the functional role of survivin was assessed in CEP plus TRAIL-mediated apoptosis, using survivin-overexpressing cells. Ectopic expression of survivin markedly blocked CEP plus TRAIL-induced sub-G1 population and PARP-1 cleavage (Figure 4D). In addition, we investigated the critical mechanism of CEP-mediated survivin downregulation. Deubiquitination enhances protein stability [21]. We examined the expression levels of several deubiquitinases in CEP-treated cells. Among them, we found that CEP decreased STAMBPL1 expression and increased USP53 expression in a dose-dependent manner (Figure 4E). Next, we investigated whether overexpression of STAMBPL1 prevents CEP-induced survivin downregulation. Ectopic expression of STAMBPL1 significantly inhibited CEP-induced survivin downregulation (Figure 4F).

### 2.5. Combined CEP and TRAIL Treatment Enhances Apoptosis in Other Cancer Cells, but Not Normal Cells

**Figure 5:**
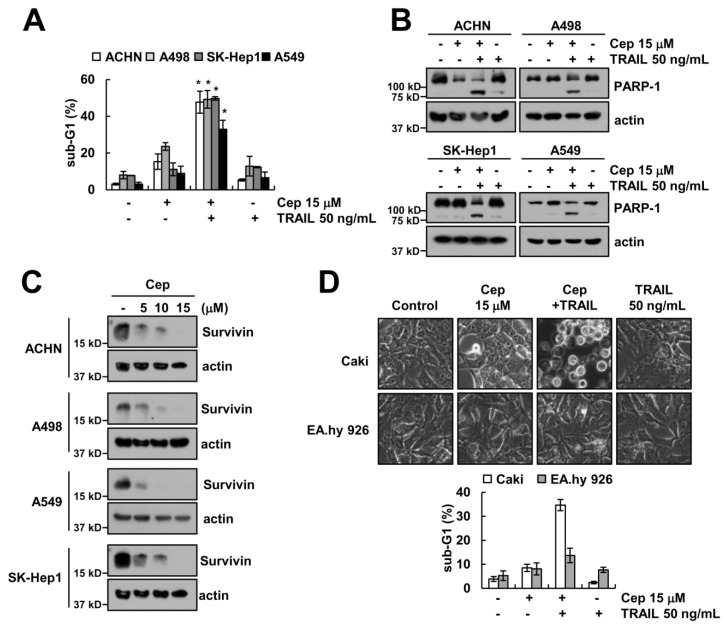
The effects of CEP on TRAIL sensitization in other cancer cells and normal cells. (**A**,**B**) Caki cells were treated with 15 µM CEP and/or 50 ng/mL TRAIL for 18 h; (**C**) cells were treated with CEP (5–15 µM) for 18 h; (**D**) Caki and normal human umbilical cells (EA.hy 926) were treated with 15 µM CEP and/or 50 ng/mL TRAIL for 18 h. Morphology of cells was detected by interference light microscopy. The sub-G1 population and protein levels were detected by flow cytometry and Western blotting, respectively. Data represent the mean ± SD of at least three independent experiments. * *p*  <  0.05 compared with the control.

Next, we investigated whether combined CEP and TRAIL treatment enhances apoptosis in other cancer cells and normal cells. As shown in Figure 5A,B, CEP plus TRAIL induced the sub-G1 population and PARP-1 cleavage in other renal cell carcinoma (ACHN, A498), hepatocellular carcinoma (SK-Hep1), and lung carcinoma (A549) cells. Furthermore, CEP also decreased the survivin protein expression levels in those cells (Figure 5C). By contrast, CEP plus TRAIL did not induce morphological change and apoptotic cell death in normal human umbilical cells (EA.hy 926) (Figure 5D).

## 3. Discussion

Tumors still show complex demeanor-like resistance to curative actions, even though many therapeutic treatments have been developed to overcome cancers. Our aim is to establish the molecular mechanisms underlying CEP plus TRAIL-induced apoptosis to meet the present demand for anticancer therapy in renal cell carcinoma. Here, we established a combined CEP and TRAIL treatment to influence apoptosis in TRAIL-resistant renal carcinoma, hepatocellular carcinoma, and lung carcinoma cells, but not in normal cells. Thus, CEP may be considered as a potent TRAIL sensitizer for cancer therapeutics.

CEP is a naturally active compound alkaloid, and it has been used as an anticancer agent for various drug-resistant cancer cells [22,23]. Antitumor activity of CEP (2–20 µM) is associated with induction of ROS generation [24], but CEP (15 µM) is not involved in ROS-mediated TRAIL sensitization in our system. We also investigated whether CEP induces endoplasmic reticulum (ER) stress marker proteins, but CEP did not induce ER stress in Caki cells.

Survivin is the smallest protein of the IAP family, and acts as an anti-apoptotic regulator through inhibition of caspase activation. The expression level of survivin is very high in cancer cells [25,26]. Survivin expression level is modulated at the transcriptional and post-translational level [27,28,29]. We found that CEP did not inhibit survivin mRNA expression (Figure 4A), but proteasome inhibitor rescued the downregulation of survivin by CEP (Figure 4C). Ubiquitination is a volatile process which is negatively regulated by deubiquitin enzymes (DUBs) [30]. DUBs can be classified into five groups: ubiquitin-specific proteases (USPs), ubiquitin C-terminal hydrolases (UCHs), ovarian tumor proteases (OTUs), Machado–Joseph disease proteases, and JAB1/MPN/Mov34 metalloenzymes (JAMMs) [31]. STAMBPL1 is one of the JAMM deubiquitin enzymes. STAMBPL1 has various molecular functions, such as functioning as a positive regulator of Tax-mediated NF-κB activation [32], but the role of STAMBPL1 is unclear in cancer cells. We found that downregulation of STAMBPL1 might be involved in CEP-induced downregulation of survivin (Figure 4F). In addition, post-translational regulation of survivin was modulated by aryl hydrocarbon receptor-interacting protein (AIP), and activation of E3 ligase by complex of XIAP-XAF1 [33,34]. Chen et al. also reported that depletion of p21-activated kinase (PAK1) enhances ubiquitin-mediated survivin degradation in pancreatic β-cells [35]. However, PAK1-specific inhibitor did not induce survivin downregulation in Caki cells), and CEP did not modulate XIAP expression (Figure 1E). Therefore, further experiments are required to identify which E3 ligases are involved in survivin degradation.

Taken together, our study suggests that CEP enhances TRAIL-induced apoptosis by degradation of survivin through downregulation of STAMBPL1 deubiquitin enzyme in renal carcinoma cells. Therefore, combined CEP and TRAIL treatment might be an attractive therapeutic strategy for TRAIL-resistant cancers.

## 4. Methods and Materials

### 4.1. Cells and Cell Culture Materials

American Type Culture Collection (Manassas, VI, USA) supplied all cell lines, and cells were cultured in Dulbecco’s modified Eagle’s medium consisting of 10% fetal bovine serum, 5% penicillin–streptomycin antibiotic, and 100 μg/mL gentamycin. R&D (Minneapolis, MN, USA) supplied the recombinant human TRAIL, z-VAD, and anti-survivin antibodies. Anti-Mcl-1, anti-STAMBPL1, anti-USP53, and DR5 siRNA was purchased from Santa Cruz Biotechnology (Dallas, TX, USA), and Cell Signaling Technology (Beverly, MA, USA) supplied the anti-PARP-1, anti-Bcl-2, anti-Bcl-xL, anti-caspase-3, anti-cIAP1, anti-c-FLIP, and anti-DR5 antibodies. Anti-DR4 antibody was obtained from Abcam (Cambridge, MA, USA). BD Biosciences (San Jose, CA, USA) supplied the anti-Bim and anti-XIAP antibody. STAMBPL1 plasmid was provided by Dr. Eek-Hoon Jho (University of Seoul, Seoul, Korea). Control (GFP) siRNA was purchased from Bioneer (Daejeon, Korea). Anti-actin antibody and all other reagents were supplied by Sigma Chemical Co. (St. Louis, MO, USA).

### 4.2. Flow Cytometry Analysis and Western Blot Analysis

Flow cytometry analysis was conducted as mentioned in our previous study [36], and we carried out Western blot analysis using RIPA lysis buffer, which was described in our previous studies [37,38].

### 4.3. Asp–Glu–Val–Asp-ase (DEVDase) Activity Assay

Caspase-3 activity was tested by obtaining cell lysates in 100 μL reaction buffer, and the measurement was performed using a spectrophotometer at 405 nm [39].

### 4.4. Reverse Transcription-Polymerase Chain Reaction (RT-PCR)

We isolated total RNA using TRIzol reagent (Life Technologies, Gaithersburg, MD, USA), and cDNA was prepared using Moloney murine leukemia virus (M-MLV) reverse transcriptase (Gibco-BRL, Gaithersburg, MD, USA) [36,40].

### 4.5. Detection of DR5 on Cell Surface

Cells that were detached using 0.2% EDTA were washed with PBS, and then suspended in 100 µM PBS including 10% FCS and 1% sodium azide. After that, cells were incubated with primary antibody (DR5-phycoerythrin; Abcam, Cambridge, MA, USA) for 1 h at room temperature. Then, the cells washed with PBS including 10% FCS and 1% sodium azide, and suspended in 400 μL of PBS for detection of DR5 on cell surface by flow cytometry.

### 4.6. Statistical Analysis

The values in all bar graphs show the mean ± SD from three autonomous samples. Statistical analysis was performed by one-way ANOVA and post hoc comparisons (Student–Newman–Keuls) using the SPSS 22.0 software (SPSS Inc., Chicago, IL, USA). *p*-values < 0.05 were considered significant.

## Abbreviations

TRAIL	TNF-related apoptosis-inducing ligand
CEP	Cepharanthine
DR5	death receptor 5
STAMBPL1	STAM-binding protein-like 1
CHX	Cycloheximide
